# Supplementary material for: Bioprospecting of soil-borne microorganisms and chemical dereplication of their anti-microbial constituents with the aid of UPLC-QTOF-MS and molecular networking approach
Source: PeerJ. 2024 Jul 18;12:e17364. doi: 10.7717/peerj.17364 (PMC11260408; doi:10.7717/peerj.17364)
Supplement: Supplemental Information 1 [file peerj-12-17364-s001.docx]

Supplementary Figures and Tables

**Bioprospecting of soil-borne microorganisms and chemical dereplication of their anti-microbial constituents with the aid of UPLC-QTOF-MS and Molecular Networking approach**

Adivhaho Khwathisi, Ntakadzeni Edwin Madala, Asfatou Ndama Traore, and Amidou Samie*

^1^Department of Biochemistry and Microbiology, University of Venda, Private Bag X5050, Thohoyandou, South Africa

Corresponding Author:

Amidou samie ^1^

University Road, Thohoyandou, Limpopo, 0950, South africa

Email address: Samie.amidou@univen.ac.za


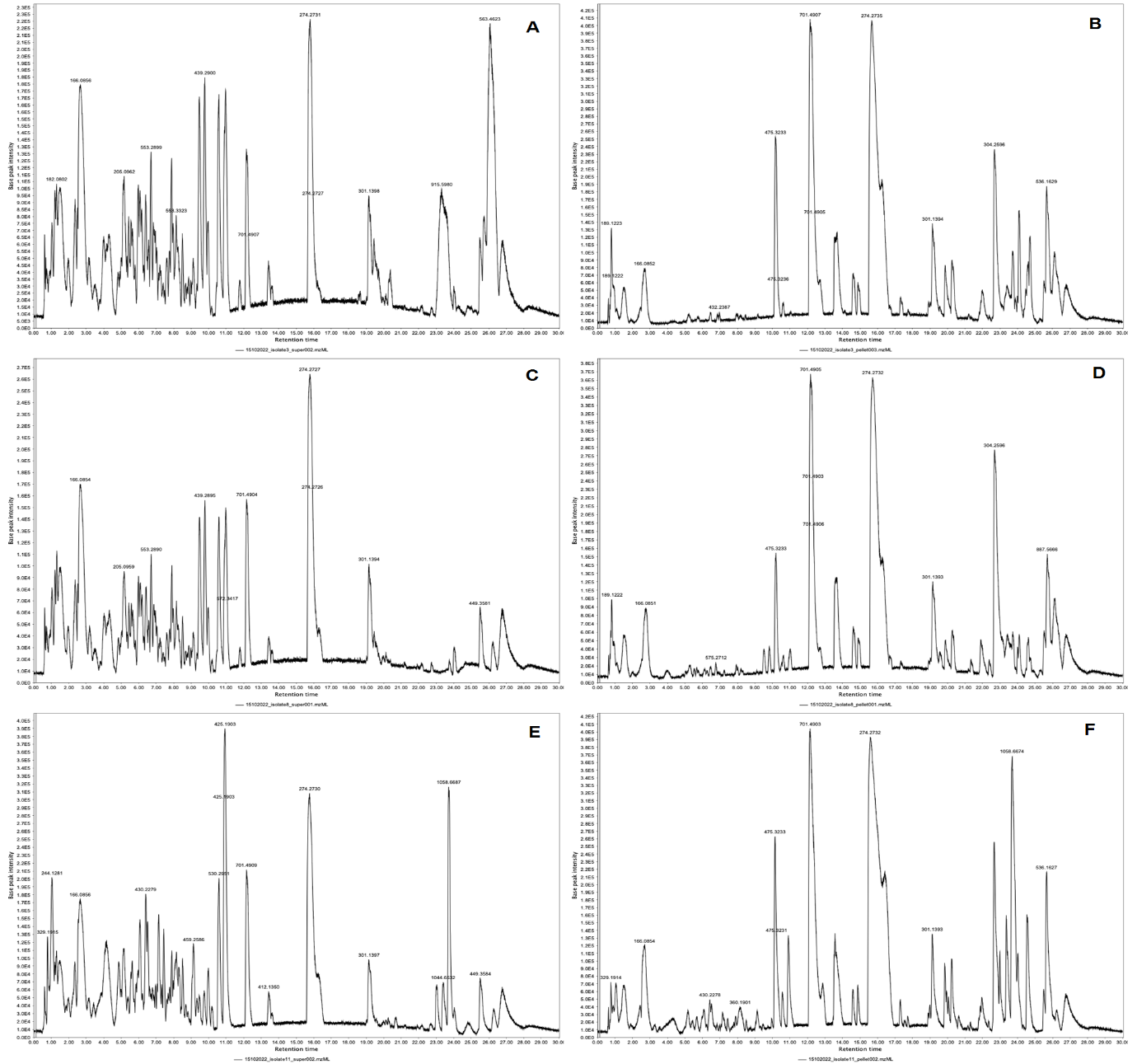
 **Supplementary Fig 1:** Representative base peak intensity chromatograms (BPC) indicating the separation of metabolites in methanol extracts of isolate 3 supernatant (A) and pellet(B), isolate 11 supernatant (C) and pellet(D), and isolate 8 supernatant (E) and pellet(F), indicating some differences in the metabolite profiles of these stains.

**
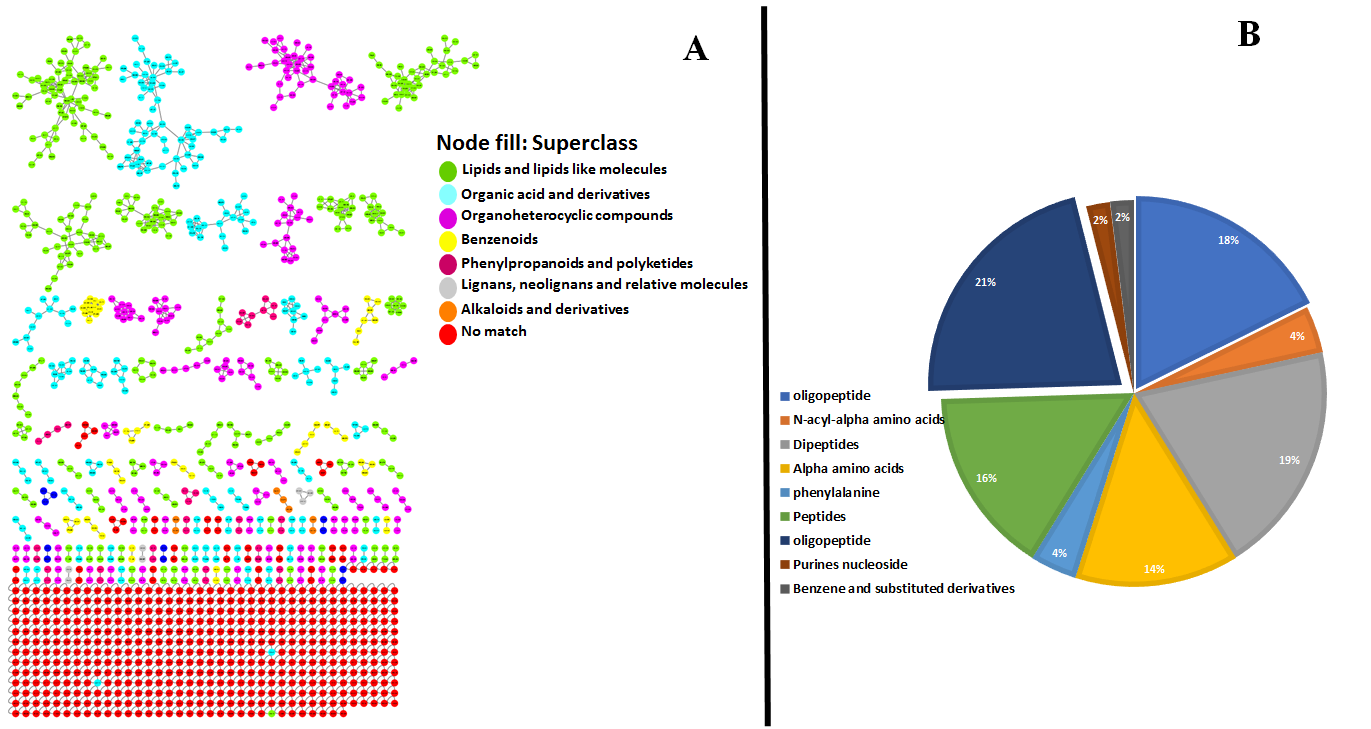
**

**Supplementary Fig 2:** Chemical classification by MolNetEnhancer of Positive ionization datasets acquired through UPLC-QTOF-MS (A), as indicated in the figure legend revealed 7 different chemical superclasses. Pie chart (B) representing the Class which were identified by MolNetEnhancer. It can be observed that bacterial strains can produce a diverse compound with wide spectra of subclasses from the chemical classification. We can also observe that the larger molecular families are mostly annotated with Lipids and lipids (Green) and Organic acids and derivatives (Blue).


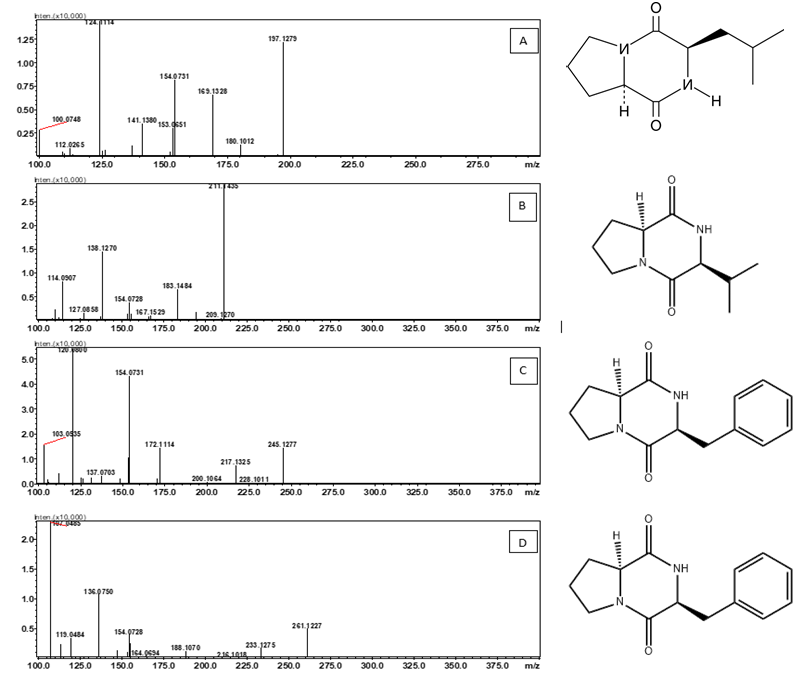
 **Supplementary Fig 3:** Typical mass spectra of the fragmentation patterns of cyclo (L-Pro-L-Val) (A), cyclo (D-Pro-D-Leu) (B), cyclo(L-Tyr-L-Pro) (D) and cyclo (L-Pro-D-Phe) (C) with their typical structures.


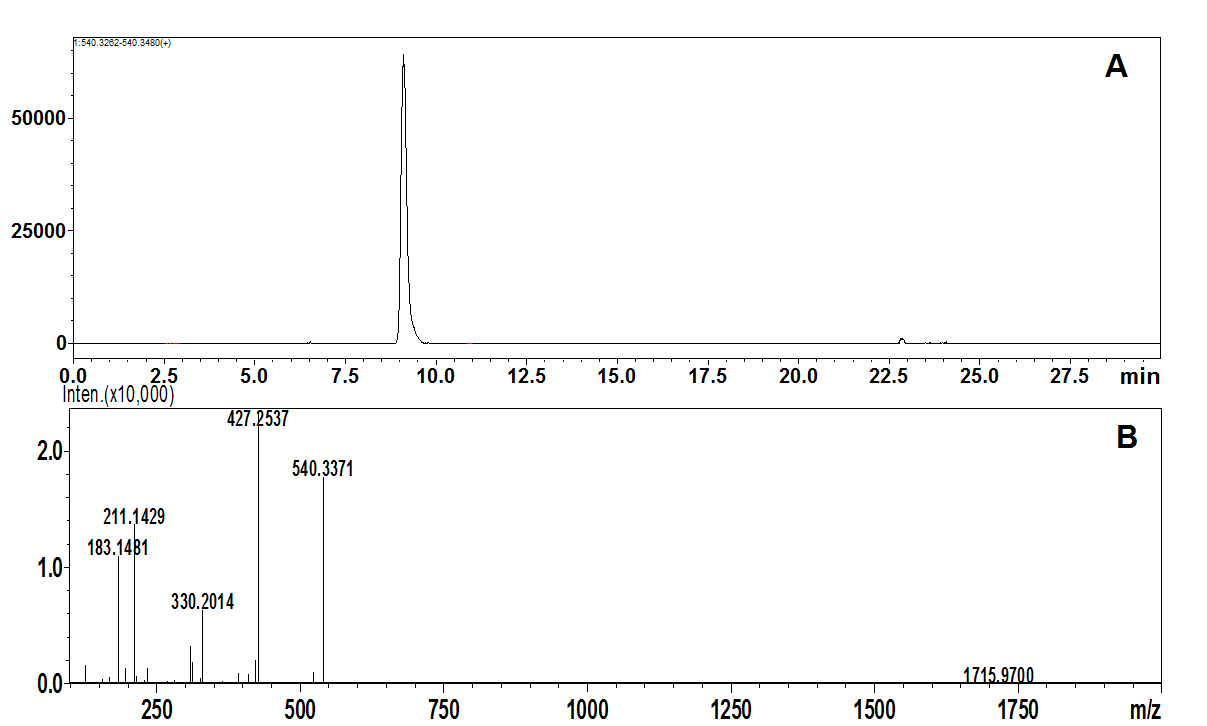


**Supplementary Fig 4:** Representative UHPLC-qTOF-MS/MS chromatogram showing single ion chromatogram of putatively identified Isarrin D (A) at *m/z* 540, with Y-axis showing peak intensity and X- axis showing retention time. Fragmentation spectra of the metabolite putatively identified, precursor at *m/z* 540 (B), with product ions at *m/z* 427, 330,211 and at *m/z* 183.


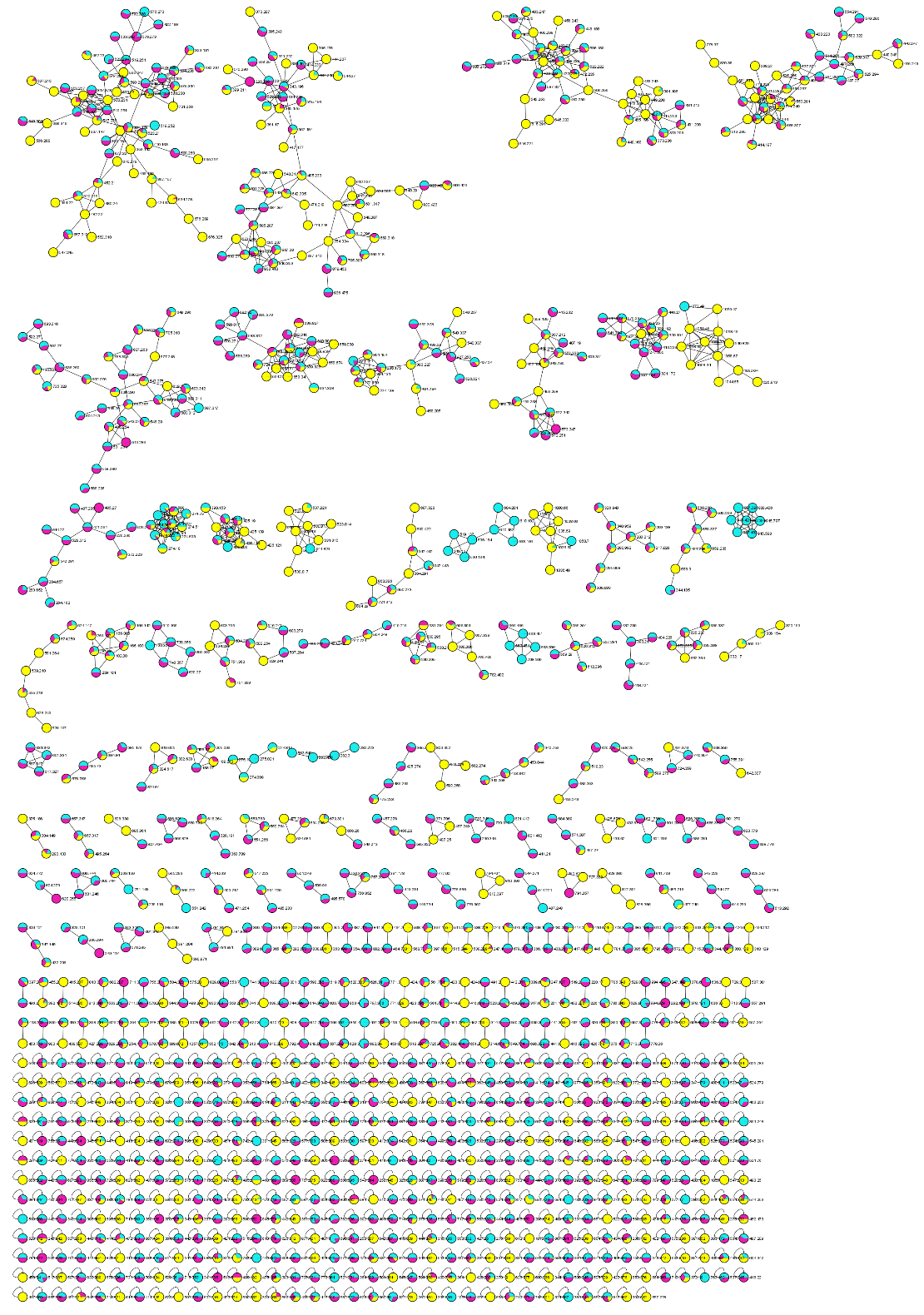


**Supplementary Fig 5:** Molecular network Analysis of antimicrobial compounds from three bacterial isolates. Blue, purple, and yellow represent the secondary metabolites from isolate 1, isolate 2 and isolate 3, respectively. Nodes in the network represent individual compounds detected in the LC-QTOF-MS.

**Supplementary Table 1:** An enhanced view of the metabolome of Bacillus strains thought GNPS spectra library matching and manual annotations. Spectral libraries searched includes Knacksack, Massbank, NIST, Human Metabolites and ResSpect.

| ***m/z*** | **Rt** | **Elemental composition** | **Fragment ions** | **Putative annotation** |
| --- | --- | --- | --- | --- |
| 116.0700 | 0.84 | C5H9NO2 | 116 | Proline |
| 118.0854 | 0.92 | C5H11NO2 | 118 | Glycine betaine |
| 182.0799 | 1.34 | C9H11NO3 | 165,136,119 | L-Tyrosine |
| 132.1010 | 1.60 | C6H13NO2 | 120120 | Leucine |
| 166.0853 | 2.76 | C9H11NO2 | 120 | Phenlyalanine |
| 229.1535 | 3.48 | C11H20N2O | 229, 183, 118 | Leucylproline |
| 342.2371 | 8.69 | C17H31N3O4 | 229, 183 | Isoleucyl-proline |
| 205.0959 | 5.15 | C11H12N2O2 | 188, 170 159, 146, 132 132, 118 | L-Tryptophan |
| 602.29 | 0.82 | C23H39N9O10 | 531, 389. 197 | Grgesp |
| 260.12 | 0.83 | C10H17N3O5 | 213, 156, 122, 110 | L-alanine, N[N(N-acetyl-L-alanine))] |
| 284.14 | 1.02 | C12H19N3O5 | 152, 135, 110 | L-proline, N[N(N-acetyl-L-alanine))] |
| 301.15 | 1.05 | C12H17N3O5 | 244, 155, 127 | L-proline, 1- [N(5-oxo-L-proly) glycyl] |
| 244.13 | 1.05 | C10H17N3O4 | 187, 155, 127 | Glycylprolylalanine |
| 247.13 | 1.09 | C10H18N2O5 | 229, 183, 118 | Valyl-glutamate |
| 269.16 | 1.21 | C12H20N4O3 | 223, 110 | Histidylleucine |
| 235.12 | 1.21 | C11H14N4O2 | 207, 162, 110 | Cyclo(his-pro) |
| 229.12 | 1.32 | C10H18N2O5 | 229, 183 118 | pyroglutamylvaline |
| 247.13 | 1.32 | C11H20N2O5 | 229, 183, 155, 138, 118, 102 | Glutamylvaline |
| 261.14 | 1.50 | C11H20N2O5 | 197, 148, 130, 102 | Leucyl-Glutamate |
| 432.24 | 1.51 | C19H33N3O8 | 319, 301, 206, 188, 170, 153 | ACMC-20lebal |
| 261.14 | 1.73 | C11H20N2O5 | 197, 148, 130, 102 | Leucyl-Glutamate |
| 471.22 | 1.86 | C19H30N6O8 | 357,244, 187 | Atrial peptide |
| 284.10 | 1.98 | C10H13N5O5 | 152, 135, 110 | Guanosin |
| 364.16 | 2.03 | C16H21N5O5 | 235, 209, 181, 156,152, 110 | TRH, free acid |
| 377.15 | 2.06 | C22H20N2O4 | 341, 241, 189 | Opreal-082153 |
| 251.17 | 2.14 | C12H18N4O2 | 223, 178, 110 | Cyclo(his-leu) |
| 281.11 | 2.48 | C13H16N2O5 | 234, 120, 103 | Phenylalanylasparic acid |
| 291.10 | 2.51 | C10H10N8O3 | 268, 255, 232,203,187,159 | CB Micro-033252 |
| 1030.66 | 23.3 | C51H89N7O13 | 917, 786,707,643,481,391,279,186 | Surfactin A (C13) |
| 1044.66 | 23.6 | C52H91N7O13 | 931, 800,707, 594, 481, 391,316,227 | Surfactin B (C14) |
| 1058.67 | 23.6 | C53H93N7O13 | 945, 814,707,594,463,320 | Surfactin C (C15) |
| 1022.67 | 23.3 | C52H91N7O13 | 909,794,685,582,441,338,227 | Surfactin C14 homolog |
| 1036.69 | 23.6 | C53H93N7O13 | 923,810,685,596,483,441,352,227 | Surfactin C14 |
| 239.103 | 1.9 | C11H14N2O4 | 165, 147, 136, 119 | Gly-Tyr |
| 1088.076 | 24.3 | C55H99N7O12 | 1086, 707,608, 477 | Pumilacidin E |
| 1078.73 |  | C56H99N7O13 | 965,852,699,568,455,227 | Pumilacidin C |
| 282.279 | 0.78 | C18H35NO | 264, 195, 177, 128 | 9-Octadecenamide, (Z) |
| 366.21 | 2.63 | C17H27N5O4 | 251,234,166,156,110 | Angiotensin1/2 |
| 369.11 | 2.69 | C16H20N2O6S | 222, 204, 186, 178, 160, 138 | Penicilloyl V |
| 260.16 | 3.01 | C11H21N3O4 | 242, 197, 132, 129 | L-isoleucine, L-glutaminyl- |
| 304.13 | 3.06 | C12H21N3O4S | 247, 155, 127 | ACMC-20mcas |
| 328.14 | 3.23 | C15H21NO7 | 310,292,264,246,178,166,143,132,120,103 | fructose-phenylalanine |
| 295.13 | 3.31 | C14H18N2O5 | 248, 120 | phenylalanylglutamate |
| 316.19 | 3.76 | C14H25N3O5 | 215,199,171,127 | threoylvalylproline |
| 227.10 | 3.90 | C10H14N2O4 | 209, 181, 139, 125 | L-proline, 5-oxo-L-prolyl |
| 325.12 | 4.31 | C19H12N6 | 296, 273, 264 | 4-(benzotriazol-1yl)-2-pridin-3-ylquinazoline |
| 302.17 | 4.73 | C13H23N3O5 | 143, 132 | Gly-Leu-Hydroxyproline |
| 223.11 | 4.75 | C11H14N2O3 | 166, 131,120,103 | Maybridge1_006768 |
| 279.13 | 4.88 | C14H18N2O4 | 232,214,158,132 | D-Proline, D-tryrosyl- |
| 188.07 | 5.15 | C11H9NO2 | 143,118,115 | indol-acrylate |
| 205.10 | 5.17 | C11H12N2O2 | 188,170,159,146,143,132,118,115,103 | Pacitron |
| 261.12 | 6.75 | C14H16N2O3 | 154, 136, 1199, 107 | Maculosin |
| 320.16 | 6.82 | C16H21N3O4 | 173, 120 | ACMC-20mguw |
| 440.25 | 6.93 | C20H33N5O6 | 229, 212 | L-proline, glycyl-L-prolyl-L-isoleucylglycyl- |
| 197.13 | 7.03 | C10H16N2O2 | 153, 133, 124 | cyclo(pro-val) |
| 555.24 | 7.06 | C24H30N1O6 | 498, 466, 247, 231, 155, 127 | opreal-_384201 |
| 389.17 | 7.22 | C20H24N2O6 | 345, 310, 267, 253, 143 | CBMicro_014004 |


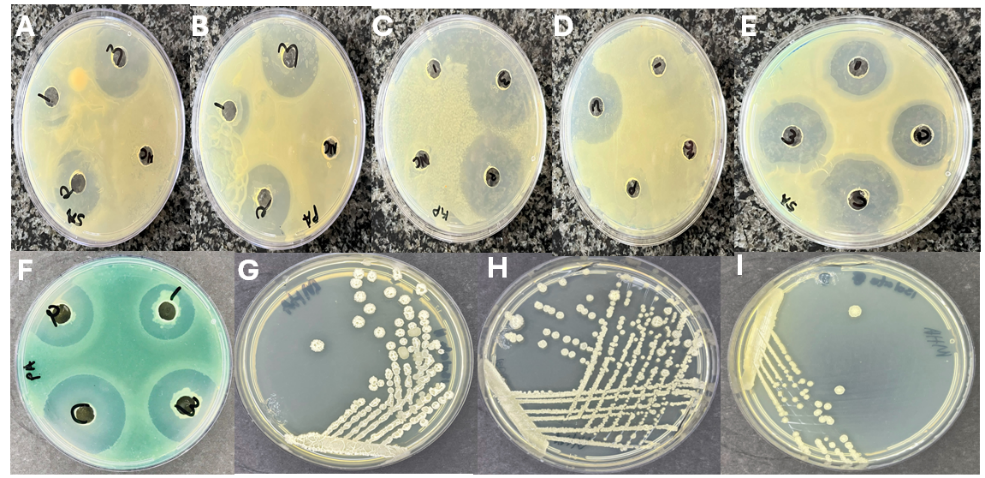


**Supplementary Fig 6:** Antimicrobial activity of bacterial isolate extracts was evaluated using agar diffusion technique against four pathogenic bacteria: (A) S. aureus, (B) P. aeruginosa, (C) K. pneumoniae, and (D) E. coli, after 48 hours of incubation. Additionally, (E) S. aureus and (F) P. aeruginosa were tested with Gentamicin (1 mg/ml) as a positive control. The isolates were streaked onto Muller-Hinton agar, labelled as (G) Bacillus subtilis (Isolate 3), (H) Bacillus pumilus (Isolate 1), and (I) Bacillus pumilus (Isolate 2).

**Supplementary Table 2:** Showing the concentration of the DNA and the corresponding A260/280 ratios measured using a nanodrop spectrophotometer.

| Sample | A260/280 | DNA conc. (ng/µl) |
| --- | --- | --- |
| Isolate 1 | 1.690 | 146 ng/µl |
| Isolate 2 | 1.542 | 130 ng/µl |
| Isolate 3 | 1.647 | 142 ng/µl |
